# IL-1*β* and IL-6 Are Highly Expressed in RF+IgE+ Systemic Lupus Erythematous Subtype

**DOI:** 10.1155/2017/5096741

**Published:** 2017-02-12

**Authors:** Yongkang Wu, Bei Cai, Junlong Zhang, Beilei Shen, Zhuochun Huang, Chunyu Tan, Carla C. Baan, Lanlan Wang

**Affiliations:** ^1^Department of Laboratory Medicine, West China Hospital Affiliated to Sichuan University, Chengdu, China; ^2^Department of Rheumatology, West China Hospital Affiliated to Sichuan University, Chengdu, China; ^3^Department of Internal Medicine, Sector Nephrology & Transplantation, Erasmus MC, Rotterdam, Netherlands

## Abstract

*Background.* Systemic lupus erythematosus (SLE) is an autoimmune disease with great heterogeneity in pathogenesis and clinical symptoms. Rheumatoid factor (RF) is one key indicator for rheumatoid arthritis (RA) while immunoglobulin E (IgE) is associated with type I hypersensitivity. To better categorize SLE subtypes, we determined the dominant cytokines based on familial SLE patients.* Methods.* RF, IgE, and multiple cytokines (i.e., IL-1*β*, IL-6, IL-8, IL-10, IL-17, IFN-*γ*, IP-10, MCP-1, and MIP-1*β*) were measured in sera of familial SLE patients (*n* = 3), noninherited SLE patients (*n* = 108), and healthy controls (*n* = 80).* Results.* Three familial SLE patients and 5 noninherited SLE cases are with features of RF+IgE+. These RF+IgE+ SLE patients expressed significantly higher levels of IL-1*β* and IL-6 than the other SLE patients (*P* < 0.05). IL-6 correlated with both IgE and IL-1*β* levels in RF+IgE+ SLE patients (*r*^2^ = 0.583, *P* = 0.027; *r*^2^ = 0.847, *P* = 0.001), and IgE also correlated with IL-1*β* (*r*^2^ = 0.567, *P* = 0.031).* Conclusion.* Both IL-1*β* and IL-6 are highly expressed cytokines in RF+IgE+ SLE subtype which may be related to the pathogenesis of this special SLE subtype and provide accurate treatment strategy by neutralizing IL-1*β* and IL-6.

## 1. Introduction

Systemic lupus erythematosus (SLE) is a typical autoimmune disease involving multiple organs [1]. SLE patients with heterogeneous symptoms might have different pathogenic factors [2, 3]. In order to better categorize SLE patients, researchers have grouped patients on genotype, laboratory tests, and clinical characteristics such as the rs329498 (CC+AC versus AA) genotype, anti-dsDNA antibody, and skin rash [4–7]. Subsequently, SLE patients were divided into different subtypes aiming to find common underlying pathogenic factors which might be helpful to unravel the disease process. These parameters are used in the daily clinical practice but are, at the same time, not distinctive enough to adapt and improve treatment for the individual patient. Cytokines play key roles in the pathogenesis of SLE [8]. Compared to healthy controls, the cytokine balance in SLE patients shifted towards a more proinflammatory phenotype [9]. It is recognized at large that both the cytokine profiles and their concentration contribute to the onset and maintenance of autoimmune diseases [10]. Therefore, treatment with agents that block cytokine mediated immune responses is an attractive option. For example, in rheumatoid arthritis (RA) patients, the disease process is successfully intervened in by anti-TNF*α* agents that interrupt the inflammatory responses [11]. Also, for the treatment of SLE, it might be attractive to block cytokine mediated responses. However, for optimal results, it is necessary to identify the dominant cytokines involved. For example, Ripley et al. [12] reported that the serum level of IL-6 is related to disease activity of SLE. Unfortunately, this observation was not confirmed by others [13–15]. One of the explanations might be that the substantial heterogeneity among SLE patients influenced the outcomes [3]. Here, we examined whether familial SLE is associated with a specific cytokine or a combination of cytokines.

Here, we reported a SLE family with 3 patients who have been described in detail before [16]. In all patients of this family, both the rheumatoid factor (RF) and immunoglobulin E (IgE) were present (RF+IgE+). RF is an autoantibody directed against the Fc portion of IgG. It is often present in the serum of RA patients. RF and IgG form immune complexes that contribute to RA process [17]. IgE plays an essential role in type I hypersensitivity, which manifests itself in immunity to parasites and various allergic diseases [18].

Until today, only a few studies have been published reporting that RF and IgE have a relationship with the pathogenesis of lupus [19, 20]. The SLE family we studied might represent an inherited SLE subtype. Based on the outcomes of the clinical laboratory tests, the patients were identified with the characteristic disease features. By our previous study results [21] and combination with the relevant literature [22], a total of nine potential pro- and anti-inflammatory cytokines were studied, that is, IL-1*β*, IL-6, IL-8, IL-10, IL-17, IFN-*γ*, interferon induced protein-10 (IP-10), monocyte chemotactic protein-1 (MCP-1), and macrophage inflammatory protein-1*β* (MIP-1*β*). We hypothesize that cytokines are differently expressed in inherited familial SLE subtype patients.

## 2. Subjects and Methods

### 2.1. Ethics Statement

This study was conducted under the approval of the Institutional Review Board, West China Hospital affiliated to Sichuan University, China. The protocol was approved by the ethics committee of our hospital.

### 2.2. Patients and Healthy Controls

**Figure 1:**
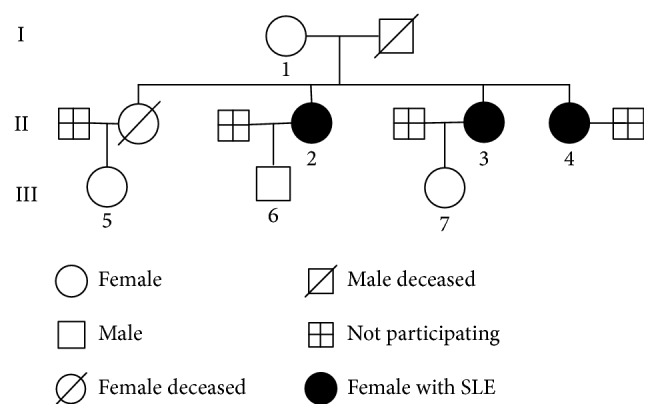
Pedigree diagram of the SLE family. Pedigree diagram of the SLE family with 3 SLE sisters diagnosed at the age of 27; the 3 patients' mother was RF positive and was suspected to have an autoimmune disease (AID). The deceased elder sister was diagnosed with clinical signs of SLE.

**Figure 2:**
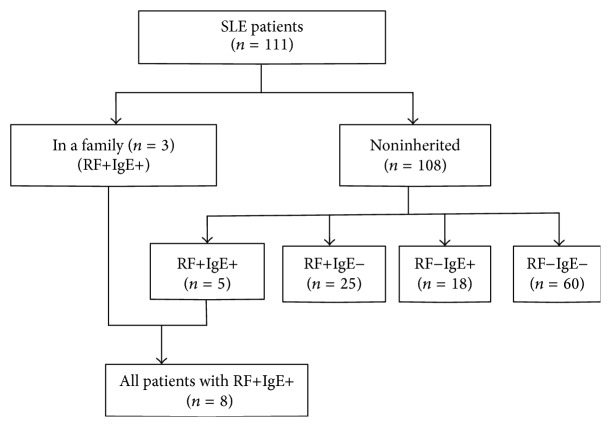
SLE patients were divided into different subgroups. All the SLE patients included 3 cases from a family with RF+IgE+; the other patients were divided into 4 subgroups based on the results combination of RF and IgE. A total of 8 SLE patients with RF+IgE+ were investigated for high expression of cytokines.* Note.* RF+ means that RF concentration is higher than the upper-limit level of our lab reference range (20 IU/mL). IgE+ means that IgE concentration is higher than the upper-limit level of our lab reference range (150 IU/mL).

Serum samples were obtained from a SLE family with 3 patients, noninherited SLE patients (*n* = 108), and healthy controls (*n* = 80). Of the SLE family, 3 daughters suffered from SLE. The first registered patient of this family was a 36-year-old female. Her younger sister (32 years old) and elder sister (38 years old) were also diagnosed with SLE. Those patients were diagnosed at the age of 27. Another proband's elder sister with clinical manifestations of SLE died (pedigree diagram is shown in Figure 1). The 108 noninherited SLE patients and 80 healthy controls were enrolled from in-patient and physical examination volunteers of the West China Hospital affiliated to Sichuan University. All SLE patients were diagnosed according to the American College of Rheumatology criteria (1997) [23]. An overview of the patient cohort is given in Figure 2.

### 2.3. Detection of RF

RF was measured by rate nephelometry method (IMMAGE800, Beckman & Coulter Company, USA).

### 2.4. Detection of IgE

IgE was measured by the microparticle chemiluminescence method (DXI800, Beckman & Coulter Company, USA).

### 2.5. Detection of Cytokines

Measurement of the cytokines including IL-1*β*, IL-6, IL-8, IL-10, IL-17, IFN-*γ*, IP-10, MCP-1, and MIP-1*β* was performed by Bio-Plex suspension chip method according to the manufacturer's instructions (Bio-Plex 200, Bio-Rad company, USA).

### 2.6. Statistics

The distribution of cytokines data was handled by median and quartile and compared by Mann–Whitney* U* test by SPSS 21.0. Pearson test was used for correlation coefficient analysis by GraphPad Prism 5.0. Probability (*P*) value less than 0.05 was considered to be significantly different.

## 3. Results

### 3.1. Demographics of Subjects

There were no significant differences in the age or gender distribution among patients in all SLE subgroups and healthy controls (*P* > 0.05) (Table 1).

### 3.2. Features of the SLE Family

Immunoglobulin (Ig), complement, and autoantibodies in sera were detected for all SLE family members. Three SLE patients in the family showed simultaneous expression of RF and IgE; one case with suspected autoimmune disease (AID) was manifested with high RF and normal IgE (data shown in Table 2).

### 3.3. Analysis of Cytokines Production by SLE Patients and Healthy Controls

In sera of all SLE patients and healthy controls, cytokines levels were determined (Table 3). The expression levels of IL-1*β*, IL-6, IL-8, IL-10, IL-17, IFN-*γ*, IP-10, and MCP-1 except MIP-1*β* were significantly higher in SLE patients than in sera from healthy controls (*P* < 0.05).

Next, the concentrations of IL-1*β*, IL-6, IL-8, IL-10, IL-17, IFN-*γ*, IP-10, and MCP-1 were compared between the SLE patients with IgE+ and those with IgE− from noninherited SLE patients (Table 4). IL-1*β*, IL-6, IL-8, and MCP-1 were significantly higher in IgE+ SLE patients than in IgE− patients (*P* < 0.05). The cytokine levels and profiles were also compared between the SLE patients with RF+ and those with RF− from noninherited SLE patients (Table 5). IL-1*β*, IL-6, and IL-10 were significantly higher in SLE patients with RF+ than in those with RF− (*P* < 0.05). Combining the results of Tables 4 and 5, we found that both IL-1*β* and IL-6 are significantly higher in SLE patients IgE+ or those with RF+. So, we investigated the IL-1*β* and IL-6 level of SLE patients with RF+IgE+. These levels were not significantly different from the 5 patients who also expressed RF+IgE+ of the noninherited group and the 3 patients of familial SLE (Table 6, *P* > 0.05). So, those 8 SLE patients with RF+IgE+ united as a big group to compare the SLE patients group without RF+IgE+. Both IL-1*β* and IL-6 were significantly higher expressed in patients with RF+IgE+ than in those without RF+IgE+ (Table 7, *P* < 0.05).

### 3.4. Association between IL-1*β* and IL-6 in RF+IgE+ SLE Patients

**Figure 3:**
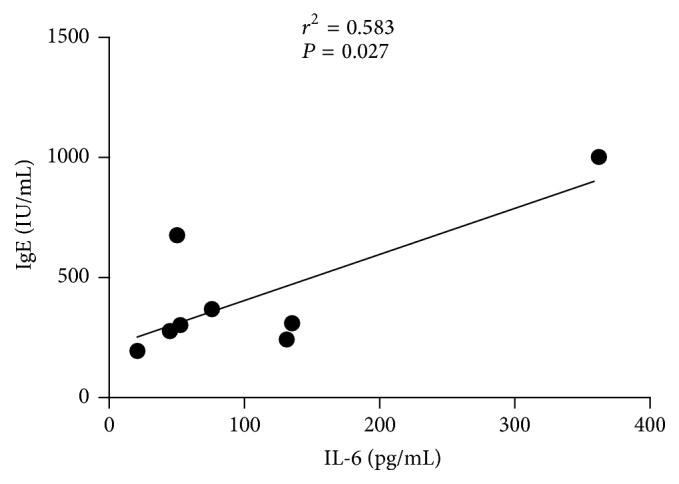
Association between IL-6 and IgE for RF+IgE+ SLE patients.

**Figure 4:**
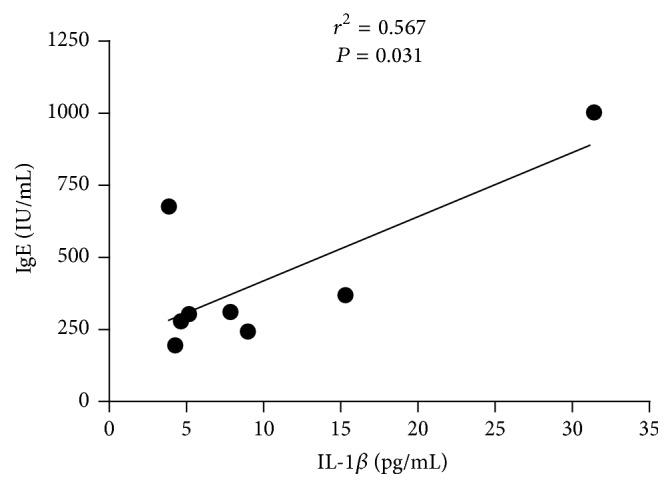
Association between IL-1*β* and IgE for RF+IgE+ SLE patients.

**Figure 5:**
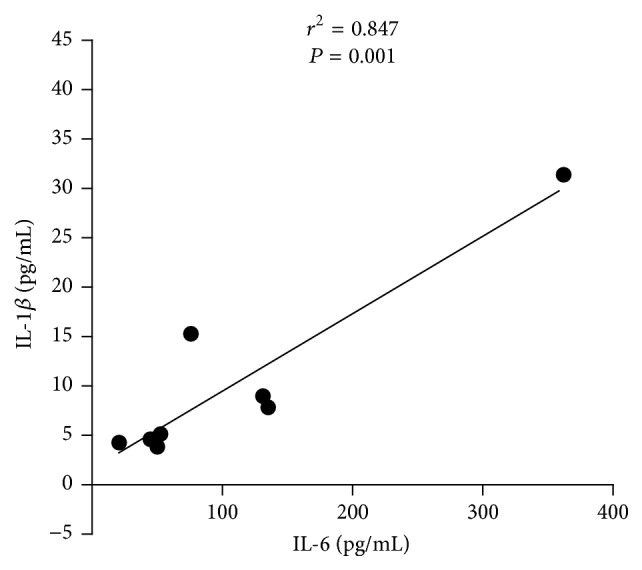
Association between IL-6 and IL-1*β* for RF+IgE+ SLE patients.

Our study showed an association between IgE and IL-6 in sera of SLE patients with RF+IgE+ (*r*^2^ = 0.583, *P* = 0.027, Figure 3); the IgE also correlated with IL-1*β* (*r*^2^ = 0.567, *P* = 0.031, Figure 4); the IL-6 level correlated with IL-1*β* levels (*r*^2^ = 0.847, *P* = 0.001, Figure 5). No correlation was found for RF with IL-1*β* (*r*^2^ = 0.077, *P* = 0.506) and RF with IL-6 (*r*^2^ = 0.000, *P* = 0.983).

## 4. Discussion

SLE has a strong heritable component up to 66% [24], and the rate of concordance in monozygotic twins is also high between 20% and 40%, while between dizygotic twins or siblings this is relatively low and about 2% to 5% [25]. However, the sibling recurrence risk ratio in SLE patients is 29-fold higher than in the general population [24]. That indicates the importance of susceptibility inheritance. Some genes such as C1q, C1r, and C1s are in very strong linkage with SLE [26]. Here, we reported three SLE patients in one family who were diagnosed with SLE at nearly the same age. This clinical observation suggests the involvement of a heritable component and one or several genes play crucial roles in the pathogenesis of SLE in this family. These patients from this family might represent a unique SLE subtype which might have an exclusive phenotype.

RF is a diagnostic criterion of RA [27], while in only 30% of the SLE patients this factor can be detected [28, 29]. Previously, studies showed that different autoimmune diseases can share the same pathogenic pathways such as gene or laboratory tests [30]. Also, RF also can be found in lupus families [19]. The family of four members including 3 SLE patients described in this paper were tested as RF positive. Therefore, we postulate that RF positive patients may represent a new SLE subtype in which RF plays a role in the pathogenesis of SLE.

High IgE level is not very often found in blood of SLE patients. Only 30% of the patients tested positive for this immunoglobulin [31]. All of the SLE patients in this family tested positive for IgE. Studies have reported that IgE leads to abnormal immune reaction which is followed by IgE production by activated B cells [32–34]. Also, activated T cells that produce IL-4, IL-5, and IL-10 provide help to B cells, which differentiate into IgE secreting plasma cells [33]. The high IgE level in a subset of SLE patients showed the involvement of the adaptive immune system in the onset and/or maintenance of this severe disease [35]. Here, some studies confirm the involvement of IgE in pathogenic mechanisms in autoimmune diseases such as lupus [20, 36]. Also, as this was found in a family with several SLE patients, it should be associated with a hereditary factor in the pathogenesis of SLE [37].

Simultaneous expression of RF+IgE+ can represent a subtype of SLE based on the heredity. In China, there are only a few families with several children, which makes this family a special group of patients to study and to discover disease related cytokine profiles. At the same time, we are aware of the limitations of our study as only 3 SLE patients in this family were analyzed which may limit the impact of our findings. Nevertheless, we believe that the outcomes of our studies provide clues for future directions of research and patient care.

Outcomes of those studies will be used to set up a diagnostic model comprising two combined potential biomarkers (RF+IgE+) to better categorize SLE patients which will also help to understand the SLE heterogeneity. From the viewpoint of heredity, if one gene mutation can cause one subtype of SLE, compared with over 100 SLE genetic loci [2], then 4.6% is high enough for RF+IgE+ SLE subtype incidence rate. We measured nine cytokines for all SLE patients. Our study showed that high levels of the proinflammatory cytokines IL-1*β* and IL-6 are present in the circulation of this unique RF+IgE+ SLE subtype. A recent study [38] reported that IL-1*β* can enhance the proliferation and differentiation of B cells to autoantibody producing plasma cells and mediate B cell apoptosis [39]. The latter is of significance as increased nuclear apoptosis enables more autoantigens exposure, leading to the production of autoantibodies to SLE [40]. Also, IL-6 is key for the activation and regulation of B cell activity [41, 42]. The study of lupus mice demonstrated that IL-6 is involved in the pathogenesis disease [43].

Here, we for the first time report that RF+IgE+ SLE patients have high serum levels of IL-1*β* and IL-6. Umare V reported that both cytokines are expressed with a high level in some SLE patients but with no results of RF and IgE [44]. These findings suggest that IL-1*β* and IL-6 may be important cytokines in this subgroup of inherited and noninherited SLE patients with RF+IgE+. In our study, no correlation was found between IL-1*β* and IL-6 with RF+. The reason may be that IL-1*β* and IL-6 can help to produce Ig, but RF that we detected was of IgM type and it shared a small part of IgM [45]. We are interested in the association with the proinflammatory cytokines IL-1*β* and IL-6. The correlation between IL-1*β* and IL-6 in our study indicated that these cytokines may collaborate in the pathogenesis of RF+IgE+ SLE subtype. Wang et al. and Apostolidis et al. also reported that both cytokines play a key role in SLE [46, 47]. Therefore, the treatment strategy of targeting the IL-1*β* and IL-6 pathway is an interesting option as these compounds might dampen disease progression.

IL-1*β* is involved in inflammatory responses and elevated levels have been found in some autoimmune diseases. Therefore, therapeutics that block IL-1*β* and/or its biological functions have been approved to treat diseases such as RA, neonatal onset multisystem inflammatory diseases, and active systemic juvenile idiopathic arthritis [48]. Recently, a new humanized antibody that binds IL-1*β* was tested with promising results which can be used in the therapeutic arsenal [49]. In addition, Prud'homme et al. proposed that an IL-1 receptor antagonist could also protect against the development of lupus by the inhibition of IL-1 effects [50]. For IL-6, Gijbels et al. also used neutralizing antibodies to treat experimental autoimmune encephalomyelitis associated with high IL-6 levels [51]. Sirukumab monoclonal antibody with high affinity for IL-6 showed potential for use in the treatment of SLE. The Phase I trial showed that sirukumab is safe and well tolerated [52]. The tocilizumab is another monoantibody directed against IL-6 receptor and blocks the downstream signal pathway by which it modulates the function of IL-6R expressing T and B cells. This agent was successfully administrated to patients with lupus-associated serositis [53], massive pericarditis, and glomerulonephritis [52, 54]. In autoimmunity, combination therapy with anti-IL-1*β* and anti-IL-6 antibody has not been tested so far. This might be a novel approach to better cure the RF+IgE+ SLE subtype patients with both cytokines highly expressed and benefit from such a strategy. This strategy was tried by Wang et al. [46] for SLE patients with high IL-1*β* and IL-6 cerebrospinal fluid levels who were found to have severe demyelinating diseases.

It is evident that confirmation in a different cohort of RF+IgE+ SLE patients is needed as our findings and conclusions are based on a small cohort of patients.

## Figures and Tables

**Table 1:** Demographics of the subjects.

Groups	Number of patients	Male/female	Age range	Mean age (mean ± SD)
SLE patients	111	0/111	14–68	35.63 ± 11.39
In the family	3	0/3	32–38	32, 36, 38^*∗*^
Noninherited RF+IgE+	5	0/5	16–40	31.40 ± 9.71
Noninherited RF+IgE−	25	0/25	14–59	37.13 ± 11.10
Noninherited RF−IgE+	18	0/18	16–55	32.39 ± 9.85
Noninherited RF−IgE−	60	0/60	14–68	35.80 ± 12.14
Healthy controls	80	0/80	14–68	35.77 ± 11.53

^*∗*^Ages of three SLE patients in the family were shown.

*Note.* RF+ means that RF concentration is higher than the upper-limit level of our lab reference range (20 IU/mL). IgE+ means that IgE concentration is higher than the upper-limit level of our lab reference range (150 IU/mL).

**Table 2:** Some laboratory data of SLE family cases enrolled in this study.

Item	Reference range	Sample number of Figure 1			
		1	2	3	4
Diagnosis	NA	Suspected AID case	SLE	SLE	SLE
RF (IU/mL)	<20.00	43.80	24.60	33.60	44.60
IgE (IU/mL)	0.10*～*150.00	141.33	278.65	1440.00	1002.87

*Note.* AID: autoimmune disease; NA: not applicable; RF: rheumatoid factor; IgE: immunoglobulin E.

**Table 3:** Serum cytokine levels of SLE patients and healthy controls.

Cytokines	SLE patients (*n* = 111) Median (25%–75%) (pg/mL)	Healthy controls (*n* = 80) Median (25%–75%) (pg/mL)	*P* value
IL-1*β*	3.86 (2.52–6.31)	1.17 (0.65–1.74)	0.000
IL-6	29.71 (19.58–50.59)	16.98 (14.24–20.74)	0.000
IL-8	101.19 (40.00–331.36)	32.12 (23.70–52.27)	0.000
IL-10	5.67 (3.95–7.66)	0.66 (0.55–3.72)	0.000
IL-17	107.54 (75.52–165.86)	90.29 (69.56–116.04)	0.003
IFN-*γ*	552.14 (444.69–629.23)	326.54 (250.89–389.31)	0.000
IP-10	7121.35 (1703.35–68843.27)	931.04 (665.78–1457.07)	0.000
MCP-1	73.39 (36.37–106.18)	57.72 (46.04–78.83)	0.021
MIP-1*β*	165.24 (65.91–530.52)	120.245 (90.69–181.78)	0.065

*Note.* The results were not normally distributed and therefore nonparametric statistics and median and interquartile range were used.

**Table 4:** Serum cytokine levels of SLE patients with IgE+ and with IgE−.

Cytokines	SLE patients with IgE+ (*n* = 23) Median (25%–75%) (pg/mL)	SLE patients with IgE− (*n* = 85) Median (25%–75%) (pg/mL)	*P* value
IL-1*β*	5.16 (3.55–15.29)	3.39 (2.22–4.92)	0.014
IL-6	48.34 (26.19–105.07)	26.19 (17.55–43.87)	0.049
IL-8	246.90 (101.19–1427.36)	73.98 (35.64–209.75)	0.005
IL-10	6.08 (5.46–8.43)	5.25 (3.61–7.64)	0.179
IL-17	130.29 (99.50–212.33)	102.83 (63.53–144.20)	0.347
IFN-*γ*	567.53 (536.75–660.14)	544.44 (395.04–613.79)	0.615
IP-10	8233.28 (3018.89–80232.70)	5783.33 (1699.61–66032.48)	0.347
MCP-1	92.83 (50.33–117.20)	65.62 (32.56–105.98)	0.019

**Table 5:** Serum cytokine levels of SLE patients with RF+ and with RF−.

Cytokines	SLE patients with RF+ (*n* = 30) Median (25%–75%) (pg/mL)	SLE patients with RF− (*n* = 78) Median (25%–75%) (pg/mL)	*P* value
IL-1*β*	4.64 (3.78–6.62)	3.34 (2.22–5.16)	0.015
IL-6	42.76 (24.43–59.88)	26.19 (17.70–40.70)	0.026
IL-8	144.38 (70.19–357.69)	73.63 (35.05–309.08)	0.115
IL-10	6.48 (4.83–8.62)	5.04 (3.37–7.13)	0.024
IL-17	116.39 (89.16–168.86)	104.51 (63.60–161.21)	0.223
IFN-*γ*	567.53 (521.38–633.10)	536.75 (404.58–623.44)	0.093
IP-10	7523.57 (1750.17–86424.87)	7423.97 (1701.48–64627.08)	0.714
MCP-1	79.75 (50.74–102.34)	67.91 (31.49–110.04)	0.297

**Table 6:** Serum cytokine levels of SLE patients with RF+IgE+ in family and in noninherited group.

Cytokines	SLE patients with RF+IgE+ in family (*n* = 3) Median (25%–75%) (pg/mL)	SLE patients with RF+IgE+ from noninherited group (*n* = 5) Median (25%–75%) (pg/mL)	*P* value
IL-1*β*	31.40; 4.63; 7.85^*∗*^	5.16 (4.07–12.14)	0.571
IL-6	362.02; 44.88; 135.26^*∗*^	52.65 (35.46–103.62)	0.393

^*∗*^Including all data.

**Table 7:** Serum cytokine levels of all SLE patients with RF+IgE+ and without RF+IgE+.

Cytokines	SLE patients with RF+IgE+ (*n* = 8) Median (25%–75%) (pg/mL)	Other SLE patients without RF+IgE+ (*n* = 103) Median (25%–75%) (pg/mL)	*P* value
IL-1*β*	6.51 (4.36–13.71)	3.55 (2.42–5.73)	0.014
IL-6	64.29 (46.21–134.27)	28.54 (18.81–47.32)	0.007
IL-8	289.13 (83.86–1288.88)	86.84 (38.4–282.23)	0.058
IL-10	5.77 (3.95–8.24)	5.67 (3.95–7.66)	0.837
IL-17	103.85 (89.18–155.74)	109.56 (67.38–168.86)	0.737
IFN-*γ*	559.84 (507.94–640.82)	552.14 (437.04–621.51)	0.444
IP-10	5268.74 (937.39–60288.90)	7249.37 (1765.77–68843.27)	0.545
MCP-1	93.91 (57.13–174.04)	70.48 (35.12–106.18)	0.202
